# Chemodenervation of the Larynx

**DOI:** 10.3390/toxins9110356

**Published:** 2017-11-02

**Authors:** Rachel Kaye, Andrew Blitzer

**Affiliations:** 1 Department of Otorhinolaryngology-Head and Neck Surgery, Rutgers New Jersey Medical School, 90 Bergen Street, Suite 8100, Newark, NJ 07103, USA; 2 New York Center for Voice and Swallowing Disorders, New York, NY 10019, USA; ab1136@aol.com; 3 Head and Neck Surgical Group, New York, NY 10019, USA; 4 Department of Otolaryngology-Head and Neck Surgery, Columbia University College of Physicians and Surgeons, New York, NY 10032, USA

**Keywords:** spasmodic dysphonia, vocal tremor, laryngeal tremor, muscle tension dysphonia, chemodenervation, botulinum toxin

## Abstract

Botulinum neurotoxin (BoNT) has existed for thousands of years; however, it was not medically utilized until investigations into its therapeutic use began in sincerity during the late 1970s and 1980s. This, coupled with the reclassification of spasmodic dysphonia as a focal dystonia, led to the use of chemodenervation for this disorder, which has since become a refined technique. Indeed, due to its safety and efficacy, BoNT has been investigated in multiple neurolaryngology disorders, including spasmodic dysphonia, vocal tremor, and muscle tension dysphonia. BoNT has been shown to be a useful and safe adjunct in the treatment for these disorders and may reduce or eliminate oral pharmacotherapy and/or prevent the need for a surgical intervention. We present the historical background, development, proposed mechanisms of action, uses, and techniques for administering BoNT for laryngeal disorders, with a particular focus on spasmodic dysphonia.

## 1. Spasmodic Dysphonia

### 1.1. History of Spasmodic Dysphonia and Botulinum Neurotoxin

Spasmodic dysphonia (SD) had long been described as a psychogenic disorder in the early 19th and 20th centuries and it wasn’t until the late 20th century that it underwent a reclassification as a focal dystonia. A fascinating historical investigation by Lorch and Whurr [1] concluded that the early confusion and misrepresentation was due to a widespread but erroneous acknowledgement of an 1864 case report as the first description of SD [2]. Indeed, Fischer reported a case of hoarse and spastic dysphonia that developed in a patient with an active typhus infection, which subsequently self-resolved when she recovered from typhus, a case which would likely be reclassified as muscle-tension dysphonia in the present day. This same case, which was termed a “Spastische Form der nervösen Heiserkeit” (spastic form of nervous hoarseness), was then reprinted in Traube’s 1871 textbook [3]. However, Mackenzie, who was a contemporary of Fischer and Traube, did report eight cases of dysphonia in 1868, which he correctly termed spasmodic with strained dysphonia [4]. His report was notably different from that of Traube and Fischer and conforms to our current clinical depiction; however, his work has been mostly overlooked by 20th and 21st century literature [5]. Instead, most credit Schnitzler with coining the term spastic dysphonia in 1875 [6] and it was this description, along with Traube’s publication, that was popularized by Arnold and Luschsinger in their 1965 textbook, where they assert that SD is a psychoneurosis [7]. Due to the futility of psychotherapy in treating this condition, general opinion eventually turned in the late 20th century [8,9] and SD was accepted and reclassified as a focal dystonia. This was aided in part by investigations of spastic dysphonia by laryngeal electromyography (LEMG) which showed that most cases actually represented a focal cranial dystonia [10,11]. Concurrently, Schantz, who had purified and crystallized botulinum toxin, in order to investigate its potential for being used as a biological weapon (for counter-terrorism purposes) for the United States (US) Army during World War II, later worked with Scott in the 1970s to pioneer therapeutic chemodenervation on monkeys [12] and humans [13] for use in strabismus and blepharospasm. Soon, the utility of botulinum toxin (BoNT) for focal dystonias was evaluated and its success led the US Food and Drug Administration (FDA) to approve the orphan drug “Oculinum” in 1989 [14]. Since then, the research on BoNT has expanded greatly and its clinical utility has followed suit. As such, we present a literature review of the topic with clinical applications provided by the senior author. A literature search was performed using PubMed until October 2017, with no limit on publication date. Eligible studies were selected by searching the keywords “botulinum larynx” and “botulinum laryngeal”, limiting articles to those published in the English language. The searches were supplemented by handsearching the bibliographies of included studies and reviews. Background information of the study, participants’ characteristics and study outcomes were collected. A meta-analysis of data was not performed.

### 1.2. Spasmodic Dysphonia Classification

SD is a focal laryngeal dystonia, which is due to disordered central motor processing that manifests as action-induced involuntary spasms of laryngeal muscles. As such, the vocal folds are normal at rest without inappropriate contractions, however during phonation there is either hyperadduction (closure) or hyperabduction (opening) of the vocal folds. Based on the nature of the spasm, SD is characterized as adductor SD (AdSD), abductor SD (AbSD), or the rare mixed SD (MSD). AdSD is most common (occurs in 87% of cases) [15,16] and manifests with hyperadduction spasms during phonation, which results in a strained voice quality as well as voice breaks. AbSD is present in approximately 13% of SD patients [16] and is characterized by a breathy, effortful hypophonic voice with abrupt termination, causing many aphonic or whispered speech segments. MSD has both components of AdSD and AbSD, manifesting as both breathy breaks and tight harsh sounds. This can at times be compensatory; however, true MSD has worsened voice outcomes when only adductor or abductor muscles are treated with BoNT, and so both muscle groups require treatment. Although most divide SD into mutually exclusive subtypes, some believe that a spectrum of adductor and abductor abnormalities exist in all SD patients and thus treatment should depend on the predominant abnormal activity [17,18]. Furthermore, Aronson et al. described that there is concomitant tremor activity found in SD patients that mimics essential tremor [19]. Indeed, Blitzer et al. found that almost 25% of SD patients have an irregular phonation-associated tremor on laryngeal electromyography (LEMG) and an additional 6% had tremor consistent with essential tremor where the tremor in question was also present during quiet respiration (see segment below) [11].

### 1.3. Spasmodic Dysphonia Diagnosis and Presentation

Obtaining a careful history is important, as some patients use sensory tricks (i.e., yawning or laughing) when initiating phonation, to alleviate the abnormal movements. Patients often describe that their symptoms wax while they are under stress and improve upon awakening in the morning or after alcohol use. Emotional responses (i.e., laughing or crying) as well as singing, are often normal while projection and speaking on the telephone are often worse [20,21]. Furthermore, attention should be paid to family history, as 12.1% of primary laryngeal dystonia patients have a family history of dystonia [21,22].

Flexible laryngoscopy allows for the examination of glottal function during vowel sounds, presence of spasms or breathy breaks, and possible tremor with connected speech. Of note, the motion abnormalities in SD are present only during specific phonation tasks, whereas they are uniformly present for all tasks with muscle tension dysphonia [23]. Also, the dystonic spasms seen in both AdSD and AbSD are intensified in magnitude by having the patient perform different vocalization tasks. Abductor spams manifest by prolonged abduction of the vocal folds and so they are most noticeable after a patient vocalizes a sound that requires vocal fold abduction. Such sounds are called voiceless consonants (i.e., /h/, /p/, or /t/) as they are produced by abducted vocal folds. When a patient has AbSD, they are able to produce a voiceless consonant, but their vocal folds have difficulty transitioning back into an adducted position. As such, the spasm is most noticeable when a patient is asked to phonate a vowel (which requires the vocal folds to be in an adducted position) immediately after a phonating a voiceless consonant. Examples of test sentences include “Harry’s happy hat” and “the puppy bit the tape” [24]. This is in contradistinction to AdSD patients, where the spasm occurs during adduction and therefore these patients have difficulty transitioning their vocal folds into an abducted position. Test sentences to provoke adductor spasms include words that begin with a vowel sound, such as counting tasks from eighty to ninety (i.e., eighty-one, eighty-two, etc.).

LEMG is a potential adjunct in the diagnosis of SD and has been studied by Hillel et al., who noted an increase in latent periods, amplitudes, and frequencies of thyroarytenoid (TA) muscle activation [25]. As mentioned below, the TA muscle is an intrinsic laryngeal muscle. Recent investigations have demonstrated an increased amplitude of TA muscle motor unit potentials (MUPs) [26] as well as increased amplitude of recruitment potentials, that are arranged in a dense bunch [27]. Furthermore, the laryngeal nerve evoked potentials (EPs) are likewise significantly increased in amplitude when compared to controls [27]. Although SD is usually diagnosed clinically, based on voice analysis and laryngoscopy, by using these characteristics it can also be diagnosed with LEMG. Currently there is gold standard diagnostic criteria for SD; however, most clinicians diagnose SD based on clinical manifestations evaluated by a detailed medical history, phonatory characteristics, and laryngoscopic examination. In fact, Ludlow and colleagues proposed a three-tier system for diagnosis, based on a screening questionnaire (self-reported symptoms), clinical speech examination, and fiberoptic laryngoscopy, which correctly classifies 97% of patients [28]. Lastly, when making a new diagnosis of SD, we urge that all patients should be evaluated by a neurologist to exclude other neurogenic diseases, such as Wilson’s disease.

### 1.4. Spasmodic Dysphonia Treatment

In the late 20th century, treatment options were limited and mostly consisted of surgical therapy or systemic pharmacotherapy, which was not particularly effective. Attempts at improving the dysphonia with speech therapy and medications used for extrapyramidal diseases were ineffective [29]. Dedo first developed the technique of recurrent laryngeal nerve surgical section to treat “spastic” dysphonia and reported his initial results in 1976, which were positive and encouraging [30]. However, Aronson and De Santo reported a 64% long-term failure rate for recurrent laryngeal nerve section in 1983 [31]. Surgical failure was thought to be due to compensatory maladaptive hyperfunction in the unoperated vocal fold. Furthermore, pharmacotherapy revealed poor results with AdSD and a 33% response rate in AbSD with the use of anticholinergics [10]. Although there is no known curative treatment of SD, it can be effectively managed. Chemical denervation via injection of BoNT is currently the gold standard of treatment and has been so for over two decades [32].

A team comprised of Dr. Blitzer, Dr. Brin, and colleagues were the first to trial the use of botulinum neurotoxin (BoNT) for chemodenervation in laryngeal dystonia and reported their results on three patients in 1986 [33] in a letter to the editor, with their formal research published in 1987 [34]. They were intrigued by the ability to chemically weaken sustained contractions in a titratable dose as opposed to the complete and permanent weakness that is obtained with surgical muscle sectioning. The initial injections were made through an electromyography (EMG) injection needle to bilateral thyroarytenoid (TA) muscles. They reported that the procedure was well tolerated, with 18 months of follow up at the time. This represented a paradigm shift in the treatment for laryngeal dystonias, as there were many advantages to chemodenervation, including lack of general anesthesia, ability to precisely target muscles with EMG guidance, graded weakening/dose titration, temporary nature of the intervention, ability to simultaneously utilize systemic medication, and cost-effectiveness. This seminal work expanded the use of BoNT chemodenervation in focal dystonia from blepharospasm and torticollis to include oromandibular dystonia, limb dystonia, tongue dystonia, hemifacial spasm, and laryngeal dystonias. Over 100 published articles to date have investigated the efficacy of BoNT injections for SD, with the combined evidence being overwhelmingly positive in favor of the value of this therapy [32]. In contrast to oral medication or laryngeal nerve section, BoNT injections into the TA muscle for AdSD have an average success rate of 90% [16,22,35,36] and as described later, additional muscles can be targeted (and overall success increased) if a patient is initially found unresponsive to traditional TA injections. See Table 1 for an expanded description of success for different treatment modalities.

**Figure 1:**
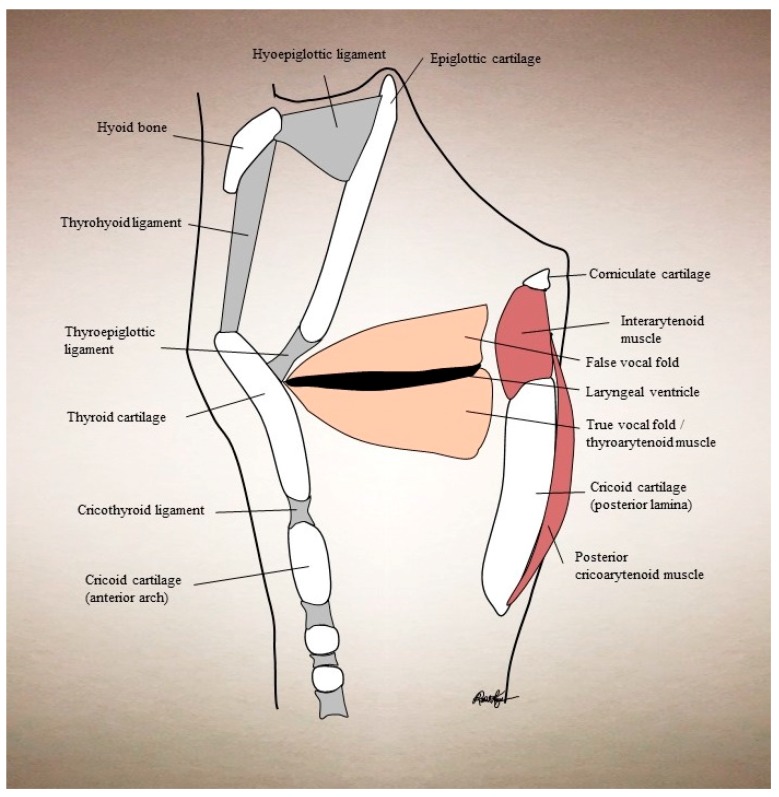
Sagittal cross-section schematic of the larynx at the midline, showing relevant bones, cartilage, ligaments, and muscles.

In understanding the treatment strategy for laryngeal disorders, one must mention laryngeal anatomy as well. Laryngeal dystonias are focal dystonias whose etiology is usually idiopathic, but can at times be secondary to other disorders [34]. They can affect a myriad of the laryngeal muscles and muscle groups. The laryngeal adductor muscles are the lateral cricoarytenoid (LCA), interarytenoid (IA), thyroarytenoid (TA), and cricothyroid (CT). The CT also acts to raise vocal pitch. The posterior cricoarytenoid (PCA) is the sole abductor muscle. Supraglottic muscles (aryepiglottic and thyroepiglottic) can also be variably affected. The strap muscles (specifically the sternohyoid and sternothyroid) can be affected with vocal tremor, as they act to depress the larynx during swallowing and phonation. The TA is the usual targeted muscle for AdSD and arises from the inner surface of the thyroid cartilage and inserts onto the anterolateral surface of the arytenoid. Its proximity to the cricothyroid membrane allows for percutaneous injection through this route (see Figure 1).

The most common injection method is by using a hollow-bore electroinjection needle, in which one can simultaneously utilize electromyographic monitoring for confirmation of placement. However, other authors have suggested direct visualization approaches, including via indirect laryngoscopy [59] and via the operative channel of a flexible fiberoptic laryngoscope [60]. These have the advantages of potentially being more familiar to the otolaryngologist and not requiring electromyographic equipment or training; however, they do require a mucosal puncture with resultant discomfort and hence the need for preprocedural topical anesthesia.

**Figure 2:**
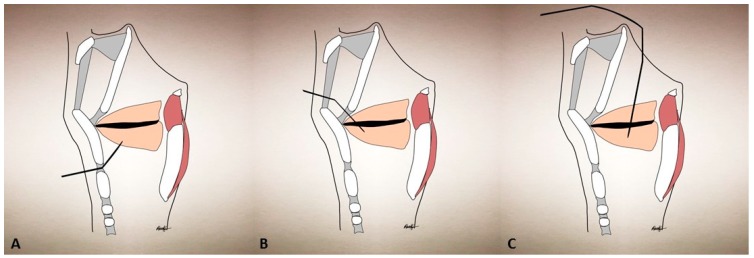
Thyroarytenoid muscle injections through different approaches: (**A**) cricothyroid approach; (**B**) thyrohyoid approach; (**C**) peroral approach.

The standard treatment for AdSD includes a percutaneous puncture of the cricothyroid membrane with a monopolar, hollow, Teflon-coated EMG needle that is directed into the TA muscle (see Figure 2). The patient is usually positioned either into an upright sitting position with a neutral head position or in a reclining position with the neck slightly extended, in order to optimize access to the cricothyroid space. We bend the needle 30°–45° superiorly and advance it superiorly and laterally through a puncture site that is just lateral to midline in the cricothyroid space; however, others keep the needle unbent. The correct location is confirmed by visualizing crisp action potentials during phonation, by prompting the patient to phonate /ē/. Otherwise, a characteristic “buzz” on the EMG would indicate that the needle has entered the laryngeal air column and instead needs to be redirected laterally. BoNT is then injected through the hollow bore of the same needle and withdrawn. Most provide an injection into bilateral TA muscles with an equal dose of BoNT; however, there are variations to this procedure as well. If a bilateral injection is to be performed, the patient is allowed to cough or swallow and then the procedure is repeated on the contralateral side. Bilateral TA injections have been the most studied treatment paradigm; however, there are some reports highlighting that unilateral injections have at least equal efficiency [61,62,63].

For dosing, we use a standard dilution of 4.0 mL of sterile saline per 100-U vial of BoNT type A (BoNT-A), which is a concentration of 2.5 U per 0.1 mL. We advise titrating this dilution to the desired potency, while attempting to maintain the volume injected per site at around 0.1–0.15 mL. This is to prevent airway obstruction from larger injection volumes. Our starting injection doses are 1–1.25 U per TA initially for AdSD with a follow up 2 weeks later, to determine the need for a titrated booster injection; however, there is a wide variation in starting doses among practitioners. Furthermore, we have noted that the average TA muscle BoNT-A doses for male and female AdSD patients varies based on gender, with males receiving, on average, 0.6 + 0.42 U and females receiving 1.3 + 1.1 U, based on a recent retrospective review of 201 patients [64].

It is important to note that although rare, patients can develop secondary resistance to BoNT-A therapy, possibly related to the development of neutralizing antibodies. This is also noted in cervical dystonia, especially for those patients who receive high doses of BoNT-A, where the incidence is estimated to be 6.5% [65,66,67,68]. The incidence of secondary resistance to BoNT-A in laryngeal disorders is not well studied, but Park et al. reported their incidence to be 8.5% in a report sampling 71 patients over a 5 year period [69]. If secondary resistance is suspected, it can be confirmed through several laboratory methods. The historical standard test has been the mouse protection bioassay, although other methods such as enzyme-linked immunosorbent assay (ELISA) and sphere-linked immunodiagnostic assay (SLIDA) exist [67,70]. Please note that these tests are not widely available and are mostly used for research purposes. As such, most cases of secondary resistance to BoNT-A in clinical practice are designated after there has been failure to respond to an appropriate or increased dose of BoNT, although this may represent disease progression and not necessarily antibody formation. A helpful clinical technique to test for secondary resistance is to inject the muscles in another area and assess the response, such as a supratherapeutic injection (i.e., 10–15 U) into the forehead (frontalis) muscle on one side, and to evaluate whether the patient develops asymmetry in forehead movement [70]; if there is no asymmetry or blunting of forehead rhytids then secondary resistance to BoNT is implied. If secondary resistance is found, BoNT type B (BoNT-B) is a safe and effective alternative [71] to BoNT-A, although it must be noted that the optimal dosing, dosing intervals, onset of action, and side effect profile differ between the two toxin serotypes. This was investigated by Dr. Blitzer in a comparative dosing study in SD patients [72] and a summary of the results are shown in Table 2. Briefly, BoNT-B requires an approximate 52 U:1 U conversion ratio when converting from BoNT-A. BoNT-B has a more rapid onset of action, shorter duration of benefit, comparable improvement in patient self-reported symptoms, and similar side effect profile (namely, dysphagia), except that it has a higher rate of autonomic side effects with dry mouth present in 9.4% of patients (a feature which approached but did not reach statistically significance). A more recent study by the same senior author [38] also found that those who received BoNT-B experienced a more rapid onset of action and shorter duration of benefit; however, they were also more likely to experience some dysphagia, compared with those who received BoNT-A, although the overall incidence was 14.2%.

**Figure 3:**
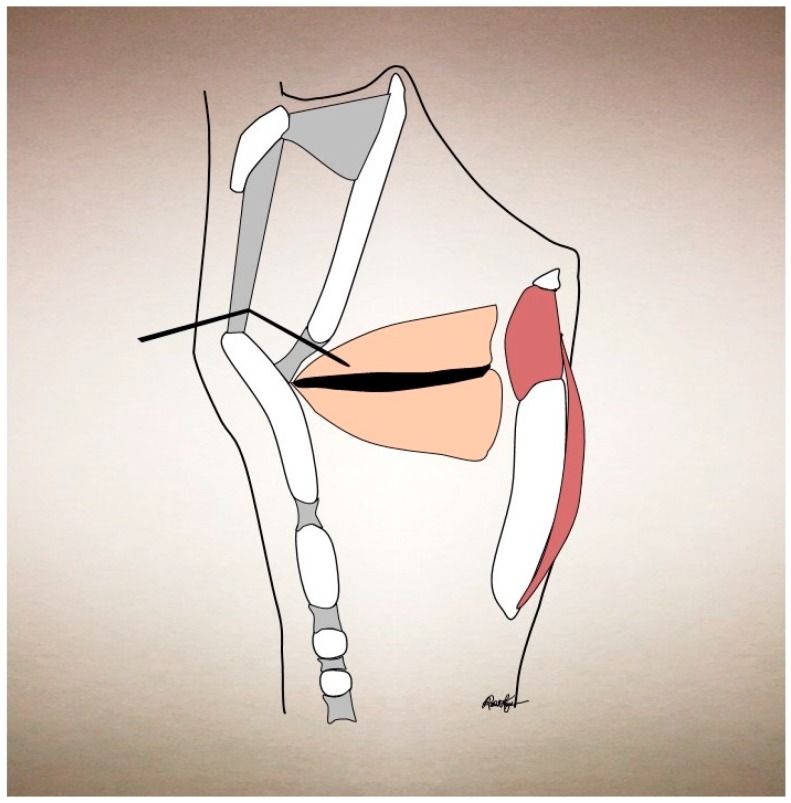
Supraglottic injection via thyrohyoid membrane approach.

Although most centers maintain AdSD patients on bilateral dosing, some prefer unilateral dosing in an effort to control symptoms of glottic insufficiency (i.e., breathy dysphonia or dysphagia to liquids). Indeed, unilateral dosing has been reported by some to provide comparable symptomatic relief with a smaller adverse effect profile [61,62,63,73,74,75]. Compensatory supraglottic hyperadduction can coexist in 25% AdSD patients and although it usually is improved with speech therapy and/or bilateral TA injections, it may persist despite these interventions and thereby curtail full improvement from standard TA injections [76]. For this, Young and Blitzer introduced the technique of targeting the supraglottis with BoNT injections, with significant improvement in these patients [76]. Simpson used the same technique, but as a primary treatment for AdSD, with the goal of reducing the initial breathy dysphonia period that can occur with TA injections [77]. This technique generally requires a higher dosing of BoNT (2.5–10 U per side) and the injection is directed into each false vocal fold. This technique also requires fiberoptic laryngoscopy to confirm placement and BoNT deposition in the submucosal space. This can be performed either through the thyrohyoid membrane with an EMG needle, or via a peroral approach. The thyrohyoid approach introduces a 27-gauge EMG needle via the thyrohyoid membrane, allowing the tip of the needle to advance just deep to the epiglottis (see Figure 3). The peroral approach utilizes a 27-gauge curved needle which pierces the mucosa and thereby requires local anesthetic, which precludes the utility of EMG guidance. Both techniques require direct visualization with a fiberopic laryngoscope to confirm the location. Despite the good results that some have experienced with this technique, there is undoubtedly diffusion of the BoNT to other laryngeal muscles, as the specific diffusion pattern of the supraglottic injection is currently unknown. It is our preference to rely upon individualized dosing into the TA muscles, with EMG guidance to successfully reduce the amount of breathy dysphonia upon BoNT onset [38] and thereby reserve supraglottic injections for those with a significant supraglottic squeeze that is not amenable to TA BoNT injections or speech therapy [76].

As mentioned above, the PCA, which is the sole vocal fold abductor, is the target muscle in AbSD. It arises from the medial and posterior surfaces of the posterior cricoid lamina and runs laterally and superiorly over the cricoid, to insert on the posterior aspect of the ipsilateral arytenoid cartilage. Given this configuration, there are two ways of achieving a percutaneous injection. Manual lateral rotation of the laryngeal framework achieves access to the posterior larynx and specifically the posterior face of the cricoid cartilage, which is the origin of the PCA. We accomplish this by placing a thumb on the posterior thyroid cartilage that is the side of planned injection. The remaining fingers are draped over the contralateral thyroid cartilage and a lateral rotation force is applied manually. The EMG needle puncture site is the lower half of the posterior aspect of the thyroid cartilage and is advanced until resistance is felt from the cricoid cartilage. The needle is then slightly retracted and the task to confirm placement is to have the patient sniff (which elicits vocal fold abduction).

**Figure 4:**
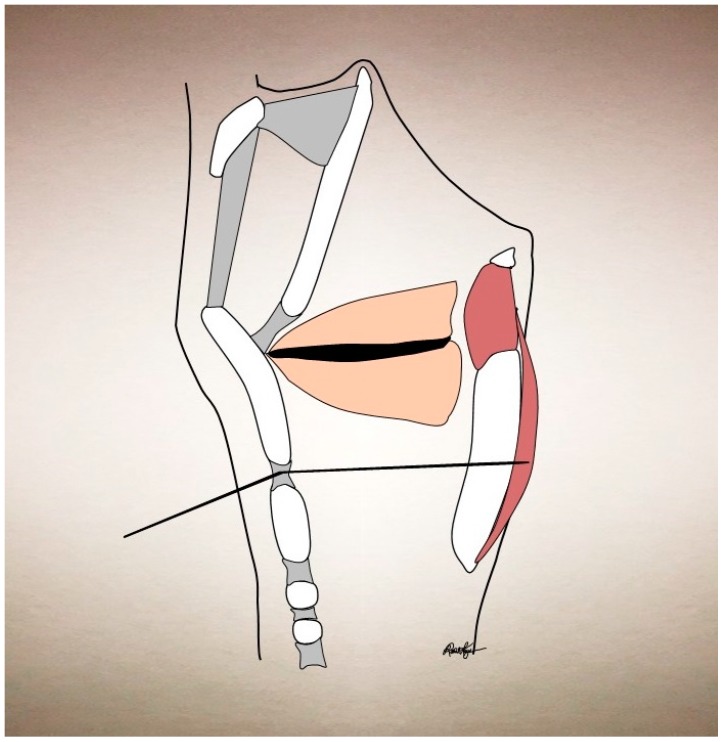
Posterior cricoarytenoid muscle injection via cricothyroid/transglottic approach.

If the patient is unable to tolerate laryngeal rotation or if it is anatomically unfavorable (thick neck or immobile larynx) then the alternate transglottic method can be used. This is performed by passing a needle percutaneously through the cricothyroid membrane, having it traverse the subglottic air column, and bore through the lateral posterior lamina of the cricoid cartilage, in order to pierce the ipsilateral PCA muscle (see Figure 4). As the laryngeal mucosa is pierced twice with this approach, topical anesthetic is usually required and can be achieved with an intratracheal injection of plain lidocaine. Although usually topical lidocaine will hinder EMG signaling, this is not as significant here, as the PCA is on the posterior side of the cricoid cartilage, sequestered from the laryngeal mucosal surface, and therefore its signaling is largely unaffected by laryngeal topical anesthetic. Correct positioning of the needle is again confirmed by asking the patient to sniff. It is important to note that the cricoid becomes increasingly ossified with age and that this may preclude use of this technique as well. Our initiation dose is typically 3.75 U in 0.15 mL of BoNT-A to a unilateral PCA muscle. We then have the patient return in 2 weeks to determine the need for a second injection to the contralateral PCA, which is determined based on symptoms and findings from a fiberoptic laryngoscopy. By staggering the injections, the risk of airway compromise is significantly decreased. Additionally, 19% of AbSD patients only require one PCA muscle chemodenervation to achieve marked improvement [78]. Whether by unilateral or bilateral injections, overall AbSD patients improve with BoNT chemodenervation to an average of 70% of normal function [78]. See Table 1 for an expanded description of success for different treatment modalities.

The success rate of TA injections for AdSD patients averages about 90% [16,22,35,36]. For patients with refractory symptoms, additional muscle groups can be targeted. LEMG can be used to determine additional target sites for refractory SD [79]. Otherwise, a trial injection of additional muscle groups can be attempted without LEMG planning. For instance, EMG abnormalities in the IA muscle of AdSD patients have been reported [25] and BoNT injections into the IA had a good response in approximately 43% of all patients and a 50% response rate for patients who were previously refractory to traditional TA or LCA injections [80]. Additionally, the LCA muscle has been reported to be dysfunctional, in addition to the TA muscle, in AdSD [79] and so this muscle can be targeted in addition to the TA muscle [36] for AdSD patients. That being said, cadaveric simulation of the diffusion pattern for TA injections shows that the extent of diffusion includes the LCA muscle 94% of the time and the CT muscle approximately 43% of the time [81]. For refractory AbSD, some have reported injecting both the PCA and CT muscles with good effect [35,78].

**Figure 5:**
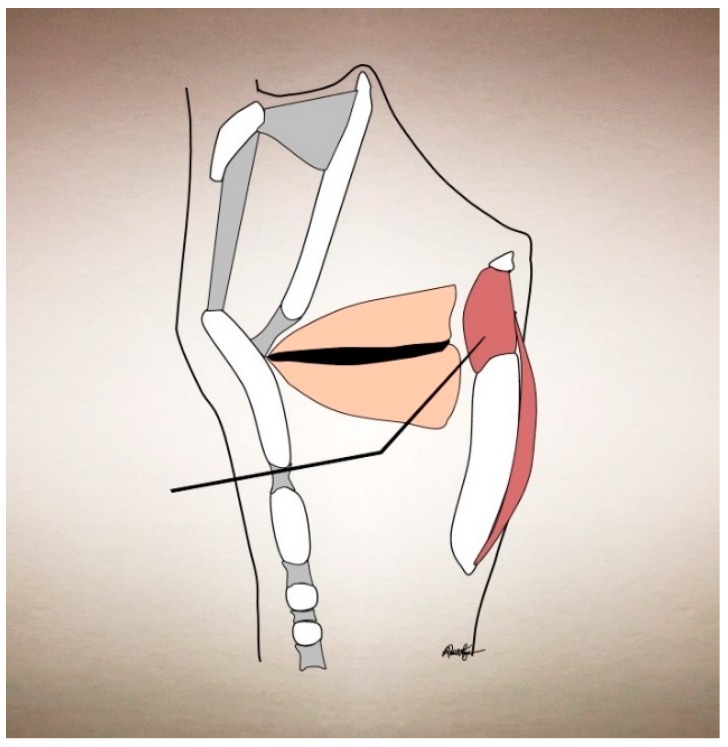
Interarytenoid muscle injection via the cricothyroid membrane approach.

IA injection can be carried out similar to a transglottic PCA injection, namely the needle is introduced through the midline of the cricothyroid membrane, passed transglottically until it pierces the muscle which is draped between the arytenoids (see Figure 5). The technique for LCA injections takes into account the muscle’s origin along the lateral ascending arch of cricoid cartilage and its insertion into the arytenoid cartilage. The injection needle is placed 1 cm lateral to midline at the level of the cricothyroid membrane, it is advanced lateral to the thyroid cartilage, passing through the CT muscle, angling posteriorly and slightly superiorly, until it pierces the LCA. The confirmation phonatory task is phonation of the long vowel /ē/. The CT injection technique is similar to that of LCA injections; the needle is inserted just lateral to midline at the level of the cricothyroid membrane/thyroid cartilage inferior border and is advanced lateral to the thyroid cartilage. Here, however, the confirmation phonatory task is asking the patient to perform a pitch glide or phonate a /ē/ as a falsetto (high pitch).

## 2. Vocal Tremor

Vocal tremor has been characterized as a separate and distinct disorder, namely, an essential voice tremor. It may be the only manifestation of essential tremor and the overall incidence of laryngeal tremor is up to 25–30% of essential tremor cases [82,83]. However, it can also coexist with SD, Parkinson’s disease, or muscle tension dysphonia (MTD). It is usually identified by perceptual analysis, as it becomes pronounced during prolonged vowel phonation, with a tremor frequency classically between 4 and 12 Hz on LEMG [46,82] and a greater amplitude than that seen in normal vibrato [48,84]. The frequency range is due to an overlap between enhanced physiologic tremor (8–12 Hz) and intention tremor (2–5 Hz) [45]. Tremor is classically present at rest and during phonatory tasks when evaluated by fiberoptic laryngoscopy and LEMG and the involuntary oscillation of the muscles involved in sound production leads to a characteristic rhythmical alteration in pitch and loudness and may even cause voice breaks [20].

A substantial proportion of patients experience relief with medical treatment. There are currently two first line therapies available: propranolol (a beta-blocker) and primidone (an anticonvulsant). Other agents include alternate beta-agonists and anticonvulsants as well as benzodiazepines and carbonic anhydrase inhibitors. It is important to note that although these medications usually do not completely resolve the tremor, they dampen its amplitude successfully in 25–55.6% of patients [49,50,51]. By comparison, BoNT has a reported 70% tremor reduction rate in the literature, and patient-reported vocal quality improvement in 56–100% of patients [45,46,47,48,85] (See Table 1). It is the senior author’s experience that the degree of acoustic improvement seen after administering BoNT for vocal tremor is not equivalent to that which SD patients experience following similar BoNT administration. This may be due in part to different etiologic pathways, extralaryngeal muscle involvement, or the fact that the tremor is usually not resolved and instead simply dampened. As such, we offer BoNT as the primary or initial treatment in those with isolated laryngeal tremor. For patients with laryngeal tremor that occurs in conjunction with tremor in other areas of the body, we consider pharmacologic treatment (with the agents listed above) as the first line therapy, and reserve BoNT as a secondary therapy if pharmacologic treatment fails.

In planning BoNT injection, it is important to characterize the subtype of laryngeal tremor on fiberoptic laryngoscopy. In general, a horizontal glottic tremor can be reduced in amplitude by injecting the TA/LCA muscles with BoNT, as described earlier. Similar to refractory SD patients, some practitioners report increased success when utilizing either LCA or TA injections and make the determination based on the predominantly dysfunctional muscle group found on EMG [86]. Maronian et al. found that 48% of their laryngeal tremor patients were maintained with LCA injections and 52% with TA injections; however, it is important to note that 28% of patients originally identified in the study were lost to follow up and may represent non-responders [86].

If a vertical laryngeal tremor is observed, then the strap muscles are targeted, specifically the sternohyoid and sternothyroid. These muscles arise from the manubrium sterni and insert upon the hyoid bone and thyroid cartilage, respectively. These are injected by utilizing an EMG-guided needle, piercing the skin at the midline at the vertical midpoint of the thyroid lamina and then angling the needle laterally to puncture the strap muscles. It is important to stay at the vertical midpoint of the thyroid lamina in order to prevent diffusion of BoNT into the base of tongue, which can produce dysphagia. If the less common abductor tremor is present, then PCA injections can be performed, using the technique described earlier for AbSD. A recently published treatment paradigm outlined the approach for coexistent vertical and horizontal laryngeal tremors, namely, that initial treatment preference is given to treat the predominant vector. If this does not bring greater than 50% relief, then both vectors are targeted. If the vertical and horizontal components have equivalent severity, then the strap muscles are targeted first, followed by staged TA injections approximately 2 weeks later. Using this systematic approach, most patients are able to have their symptoms well-controlled [46].

If the tremor exists in other locations within the larynx/pharynx (i.e., pharyngeal or tongue base), it may not be able to be targeted with BoNT and systemic therapy is advised. A recent prospective study, comparing the efficacy of BoNT to propranolol therapy, revealed that some patients preferred BoNT therapy and overall ranked a significant improvement in their voice-related quality of life after receiving BoNT. However, the enrolled patients were those who were planned for, or had already received, BoNT therapy in the past and so may have self-selected [51]. No direct comparison between BoNT and pharmacotherapy for all-comers, or one that defines the laryngeal subtypes of tremor involvement, currently exists. Again, the more diffuse the tremor, in terms of laryngeal and pharyngeal muscle involvement, the less likely BoNT will be able to be used as a monotherapy, especially if the involved sites include the pharynx or the tongue base.

## 3. Muscle Tension Dysphonia

Muscle tension dysphonia (MTD) is another functional disorder caused by hyperfunctional laryngeal musculature. Proposed etiologies include maladaptive compensatory behavior following an upper respiratory infection, vocal fold pathology, increased muscle tone secondary to reflux, high vocal demands, and psychogenic issues [52,87]. Fiberoptic laryngoscopies show significant supraglottic tension and squeeze across all phonation tasks that is not present during respiration. Unlike SD, the amount of supraglottic squeeze does not vary based on phonation task and instead provides a constant hoarse dysphonia. Treatment has generally focused on voice therapy with good results in most (See Table 1) [52]. However, non-responders have been treated with a range of techniques, including lidocaine [88,89], surgery to excise the false vocal folds [90], and false vocal fold or supraglottic injections. Supraglottic injections have been discussed in case reports and retrospective studies with good effect for refractory cases and although some were able to convert to normal phonation with a single injection, most patients were able to transition to permanent effects upon eventual adoption of voice therapy techniques [52,53,54]. Of note, although the technique for supraglottic injection is the same as described for supraglottic injection for AdSD, the doses reported in the literature are higher, ranging from 30–45 U per side.

## 4. Side Effects and Complications

As there is no standardized dosing and dosages are tailored to each patient, one must be knowledgeable of unwanted side effects which increase with dosing. Chang et al. [72] noted that there is a likely an individual threshold dose and that dose amounts above this increase side effects, without providing significant benefit. BoNT injections into the TA or LCA muscles can result in breathiness, dysphagia, and/or aspiration. A recent study of 903 customized BoNT treatments [38] found that breathiness was experienced in 50.9% of all injections, with an overall mean duration of 20 days. Of note, such breathiness does not necessarily equate to decreased perceived function, as only 28.5% actually reported decreased perceived voice function; many patients reported that although the voice could become breathy, this was often counterbalanced by the increasingly smoother speech compared with nontreatment. There was no difference in the incidence of breathiness based on toxin subtype, but there was a significant difference in the duration of the breathiness, with patients who received BoNT-B experiencing longer breathiness than the BoNT-A group. They also found that the incidence of dysphagia to liquids was 14.2% (mean duration 1–2 weeks). Dyspnea or breathlessness, while speaking, was reported in 2% of treatments, with a mean duration of 12 days. This likely represents glottic incompetence from weakened adductor musculature. In patients who received either BoNT-A or BoNT-B, a higher dose was associated with the development of breathiness; however, only those who received BoNT-B had a correlation with a higher dose and length of breathiness.

BoNT injections into the PCA muscle may result in dyspnea, a sensation of throat tightness, and/or airway compromise. However, Strong et al. [91] have shown that bilateral simultaneous PCA injections are safe and Klein et al. [43] reported that this improves results and patient satisfaction.

## 5. Conclusions

Botulinum neurotoxin can be used for a wide array of techniques and indications, in order to alleviate laryngeal disorders. It has withstood the testament of time and can greatly and tangibly affect how patients communicate and interact with the world. Laryngeal electromyography is useful in determining the muscular targets as well as guiding correct injection localization. A dosing schedule and pattern that is tailored both to individual patient response and to the disorder in question, results in significant improvement in symptoms for most. As research continues, more uses may be found and therapies can be further customized to each patient.

## Figures and Tables

**Table 1:** Expanded description of vocal improvement and adverse effects for different treatment modalities and laryngeal disorders. Surgeries undertaken for AdSD include thyroplasty and selective laryngeal adductor denervation-reinnervation. AdSD = Adductor spasmodic dysphonia, AbSD = Abductor spasmodic dysphonia, BoNT = Botulinum neurotoxin, BoNT-B = Botulinum neurotoxin type B, TA = thyroarytenoid muscle.

Condition	Treatment	Success	Side Effects
**AdSD**	BoNT	90% [16,22,35,36]	25–28.5% Breathy dysphonia
			10–14.2% Dysphagia to liquids
			9.4% Dry Mouth (BoNT-B only)
			2% Dyspnea or breathlessness
			<1% Local pain, bruising, or itch [37,38]
	Surgery	59–69% [39,40,41,42]	Relapse of symptoms [39]
	Pharmacotherapy	Anecdotally low [29]	Not reported
**AbSD**	BoNT	89% [43]	6% Dysphagia to solids (mild)
			2% Exertional wheezing [37]
	Pharmacotherapy (Anticholinergics)	33% [10]	Dry mouth, constipation, urinary retention, defective pupillary accommodation, confusion [44]
**Vocal Tremor**	BoNT	56–100% [45,46,47,48]	53% Mild hoarse dysphonia (TA injection)
			0% Strap muscle injection [46]
	Pharmacologic (Primidone)	25–54% [49,50]	73% Overall:
			30% Fatigue
			13% Nausea
			10% Unspecified
			7% Dizziness
			7% Headache
			7% Disequilibrium [49]
	Pharmacologic	55.6% (any improvement)	25% Dizziness or gastrointestinal distress [51]
	(Beta Blocker)	33% (significant improvement) [51]	
**Muscle Tension Dysphonia**	BoNT	83–100% [52,53,54]	50% Dysphagia to liquids (mild)
			32% Breathy dysphonia
			16% Tongue paresthesia [52]
	Speech therapy	100% [55]	44–65% Noncompliance rate [55,56,57,58]

**Table 2:** Comparison of BoNT-A and -B subtypes, based on findings by Dr. Blitzer in his comparative dosing study [72]. Statistically significant differences are highlighted with a * and bolded text.

BoNT Serotype	Conversion Factor	Onset of Action *	Duration of Benefit *	Autonomic Side Effects	Mean Self-Reported Symptom Improvement (0–100%)
Type A	1 U	**3.2 days**	**17 weeks**	None	89%
Type B	52.3 U	**2.09 days**	**10.8 weeks**	Dry mouth	85.4%

## Abbreviations

AbSD	Abductor spasmodic dysphonia
AdSD	Adductor spasmodic dysphonia
BoNT	Botulinum neurotoxin
BoNT-A	Botulinum neurotoxin type A
BoNT-B	Botulinum neurotoxin type B
FDA	Food and Drug Administration
EMG	Electromyography
EP	Evoked potential
LEMG	Laryngeal electromyography
MSD	Mixed spasmodic dysphonia
MUP	Motor unit potential
SD	Spasmodic dysphonia
TA	Thyroarytenoid
